# Androgen-Mediated Anti-inflammatory Cellular Processes as Therapeutic Targets in Lupus

**DOI:** 10.3389/fimmu.2020.01271

**Published:** 2020-06-23

**Authors:** Jessica M. Jones, Trine N. Jørgensen

**Affiliations:** ^1^Cleveland Clinic Lerner College of Medicine of Case Western Reserve University, Cleveland, OH, United States; ^2^Department of Inflammation and Immunity, Cleveland Clinic, Lerner Research Institute, Cleveland, OH, United States

**Keywords:** SLE, lupus, androgen, MDSC, pDC

## Abstract

Systemic Lupus Erythematosus (SLE), among many other auto-immune diseases, is known to be more prevalent in females than in males. This observation has served as the foundation for studies into how sex hormones may interact with the immune system to either drive or inhibit immune activation. Early studies using castration in lupus mouse models showed the potential protective effect of testosterone against lupus development. These studies were later corroborated by observational studies in lupus patients, who upon treatment with testosterone therapy, displayed decreased disease burden. However, there are numerous limitations to treating (especially female) lupus patients with testosterone. Thus, identification of testosterone-targeted cellular and molecular mechanisms affecting immune activation is an attractive target for lupus treatment in the future. Recent studies have examined the effects of androgens on the activation of anti-inflammatory processes. As such, immunoregulatory cell types including myeloid-derived suppressor cells (MDSCs) and regulatory T and B cells have been shown to be susceptible to manipulation by sex hormones. Here, we review studies of SLE and lupus-like disease in which testosterone or testosterone-derivatives were used to skew an ongoing immune reaction toward an anti-inflammatory state. Via evaluation of both clinical studies and immunologic models we propose new areas for research with the goal of identifying testosterone-driven anti-inflammatory mediators suitable for therapeutic targeting in patients with lupus and other autoimmune diseases.

## Introduction

SLE is an autoimmune disorder which may target multiple organs, including skin, joints, and kidneys. It has an annual incidence of 7.2 per 100,000 individuals a year in the United States (1). Current standard disease management involves the use of glucocorticoids initially, with added maintenance immunosuppressive therapy as needed. These options, however, are broadly immunosuppressive, have significant adverse effects, and do not directly target the cause of lupus.

The pathogenesis of SLE is multi-factorial with genetic as well as environmental factors implicated. Although men with lupus have been shown to have a worse course of disease than women, women are much more predisposed to getting lupus than men, with a 9:1 female to male ratio among patients. Interestingly, this ratio is the highest during reproductive years, when sex hormone levels are the highest. Moreover, disease incidence peaks in women during their reproductive years (30–50 years), while in men there is a later peak at ages 50–80 years (1). This female predisposition is poorly understood in its relation to the pathogenesis of lupus, but suggests that sex hormones play a role in disease initiation or progression. We and others have previously reviewed how sex hormones interact with the immune system in a variety of ways [reviewed in (2, 3)], however our understanding of their specific role in lupus pathogenesis remains limited. Thus, a better understanding of the relationship between sex hormones, cellular and molecular targets of sex hormones, and lupus pathogenesis may lead to new targeted therapies. In this review we will focus on clinical and laboratory studies evaluating the role of androgens in SLE and mouse lupus-like disease (lupus); highlighting potential areas of further study to improve SLE therapies, particularly within testosterone's role as an immune regulator.

## Androgen Levels and Manipulations in SLE

Early studies of sex hormone levels in SLE patients showed that female patients with active disease expressed decreased levels of androgens, including testosterone, androstenedione, dehydroepiandrosterone (DHEA), and dehydroepiandrosterone sulfate (DHEAS) (4, 5). Decreased levels persisted even after standard of care treatment and the induction of remission (6), suggesting that low levels were not a result of disease, but intrinsic to the patient population. Additional studies of testosterone therapy in SLE patients suggested a benefit from androgen therapy to disease severity (7–9), however a subsequent study failed to show improved disease activity, quality of life or sexual functioning in women with mild/moderate lupus (10). More recently, two larger studies using DHEA at a dose of 200 mg in women with SLE found a higher percentage of patients showing stable or improved disease within the DHEA group compared to the placebo group (11, 12). While reasons for the discrepancy in these results remain unclear, it should be noted, that steroid use has been identified as a potential confounder when assessing the efficacy of testosterone (13, 14). Thus, further analyses evaluating the clinical efficacy of testosterone and DHEA in SLE patients as a function of steroid use are needed to establish whether such treatments should be more widely offered.

In men with SLE, androgen levels have also been shown to be reduced (15–18), and while an early study showed no evidence of hypogonadism or androgen deficiency (15), a more recent study showed that hypoandrogenism was present in a subset of males with lupus (16). Circumstantially, treatment of male SLE patients with 19-norandrostione resulted in increased serum estrogen levels—and reduced testosterone levels—and elevated serum anti-dsDNA antibodies (18), supporting a correlation between low androgen levels and elevated autoantibodies. Interestingly, a separate study comparing testosterone levels in different patient groups with other chronic diseases also showed reduced levels across all study participants, raising the possibility that low levels of androgens are a result of the chronic inflammatory milieu in all patients and not just in lupus (17).

Finally, sex hormones are altered among transgender patient on hormone therapies. A few case reports exist of male-to-female transgender patients who developed lupus following the use of feminizing sex hormones (19–21), and a single case report of a female-to-male transgender patient, reported an established subacute cutaneous lupus erythematous prior to hormone therapy with resolution of symptoms following androgen replacement therapy (ART) (22). To our knowledge no reports have been published supporting resolution in male-to-female patients or lupus development in female-to-male patients, further supporting a protective effect of androgens and an exacerbating effect of estrogens. In summary, most studies suggest a possible therapeutic role for androgens themselves, although unwanted side effects have prevented further development of such treatments. Further identification of specific cellular and/or molecular targets of testosterone within the immune system may however represent a promising area for development of future SLE therapies.

## Androgen Manipulation in Animal Models of Lupus

Given the observations about sex hormones and lupus disease progression in SLE patients, hormone manipulation studies in animal models of lupus have been carried out, primarily using NZB/NZW F1 mice, which have a female predominance for SLE-like disease. These studies, showed a protective role for testosterone in mouse lupus (and a disease promoting role for estrogens), providing a model system in which the cellular targets and mechanisms of action of testosterone can be further studied [reviewed in (3)]. As research with this and other animal models has continued, a number of androgen mediated pathways have emerged as potential future therapy targets, particularly among regulatory cell populations and their activation by androgens.

## Regulatory Cells as Targets of Androgens?

### Regulatory T Cells (Treg)

**Figure 1:**
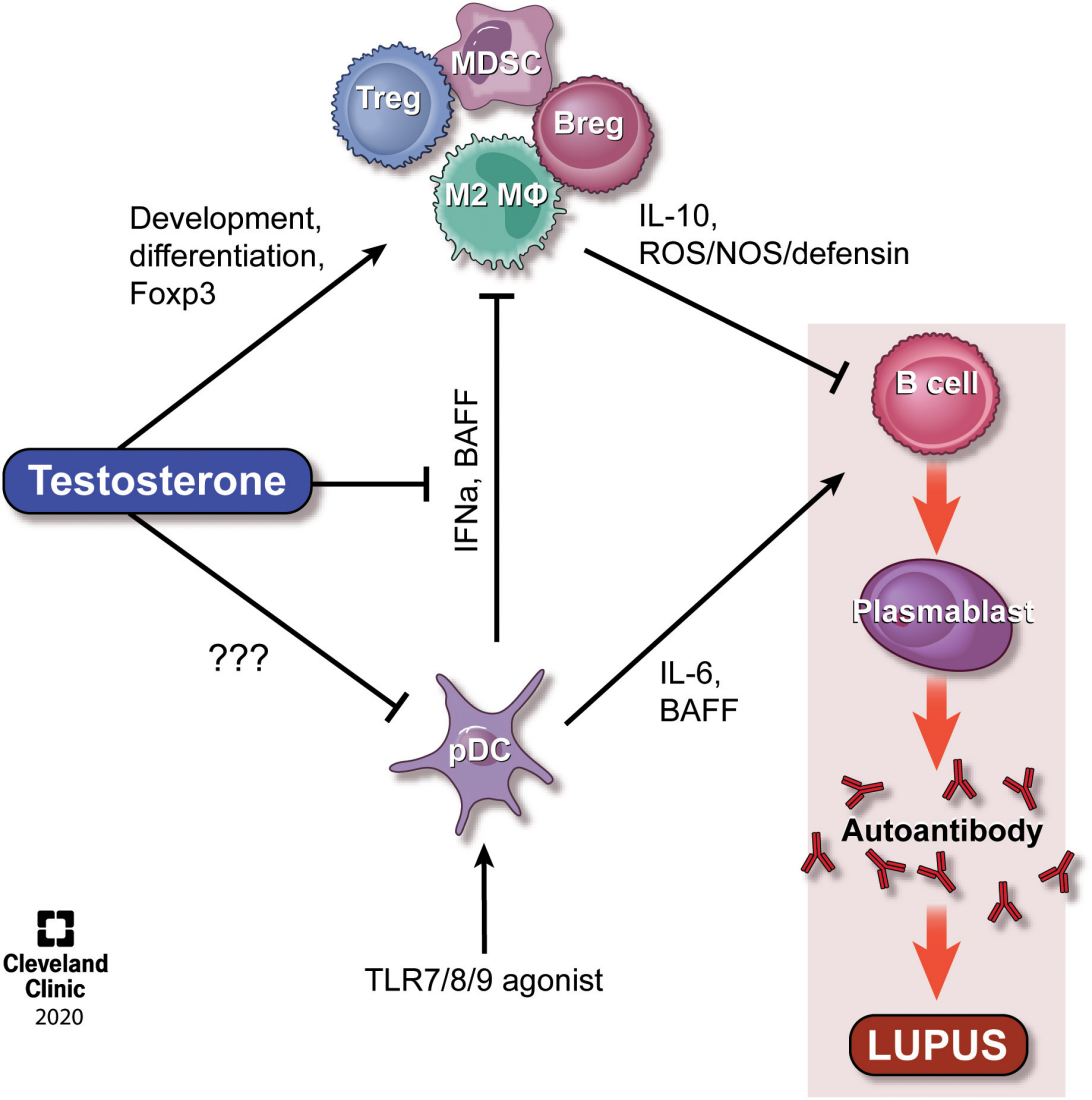
Model of the effect of testosterone on regulatory cells and the opposing effects of pDCs. It is well-established that TLR-stimulated pDCs secrete IFNα, IL-6, and BAFF, all of which actst to promote immune activation and lupus pathogenesis. Testosterone exert direct effects on the development of MDSCs and Tregs, the latter via regulation of Foxp3, and indirect effects on M2 macrophages and Bregs, potentially via regulation of BAFF. The balance between testosterone and pDC/IFNα levels represent an interesting area for therapeutic targeting in SLE. Please see the text for additional details. Reprinted with permission, Cleveland Clinic Center for Medical Art & Photography 2020. All Rights Reserved.

Regulatory T cells are represented by the natural thymic Tregs and a population of induced Tregs (iTreg) (23). Support for an association between testosterone and Tregs recently came from a study of children, in which it was found that 8 years old boys with higher levels of cord blood DHT levels at birth expressed increased levels of Tregs as compared with boys with lower levels of cord blood DHT and girls (24). In animal models, however, the literature discussing direct effects of androgens on Tregs is sparse and inconclusive. For example, on one hand it has been shown that testosterone directly affects the expression level of Foxp3 via an androgen-receptor binding motif in the proximal *Foxp3* promoter (25), while on the other hand, gonadectomy of male mice in models of virus-induced myocarditis and autoimmune hepatitis resulted in increased or no changes in Tregs, respectively (26, 27). In SLE, reduced levels and functions of Tregs have been reported in two independent studies (28, 29), while a third study surprisingly showed elevated levels of Tregs (30). Interestingly, the latter study also showed that IFNα production from SLE-derived antigen-presenting cells (APCs), but not from healthy control APCs, was responsible for inhibiting Treg functionality (30), suggesting that Treg abnormalities may be a result of elevated IFNα levels and the chronic inflammatory environment of SLE patients (see Figure 1). Therapeutically, adoptive transfer of Tregs in lupus has been investigated. A single lupus patient was treated with autologous Tregs, and the treatment resulted in increased Tregs at cutaneous inflammatory sites, as well as a shift from a Th1 to Th17 response (31). While results from only one patient are difficult to draw any conclusions from, it does support a role for investigating Tregs within the pathogenesis of lupus.

### Regulatory B Cells (Breg)

B cells are known to have a number of actions within the pathogenesis of lupus, most notably the production of autoantibodies. However, a subset of B cells known as Bregs play a suppressive role, mainly through the actions of IL-10 and TGF-β and have recently emerged as a focus within lupus (32). In healthy individuals, Bregs have been found to suppress the differentiation of Th1 cells following CD40 stimulation in an IL-10-dependent manner (33). Although Bregs have been found at increased levels in patients with SLE (34), it has also been reported that SLE Bregs are unable to suppress Th1 differentation, and have decreased capacity to produce IL-10 when stimulated with CD40 (33) and TLR9 (35). Interestingly, this dysregulation of Bregs in lupus may be driven through IFNα produced by pDCs, promoting plasmablast differentiation while suppressing Breg differentiation (36) (see Figure 1). Of note, recent drug trials in lupus included testing of compound BT063, a monoclonal humanized anti-IL-10 antibody; tested for safety and tolerability. The study met its primary endpoint for safety and tolerability, and additionally showed early signs of efficacy (35). This choice of target is interesting, given the immunosuppressive actions of IL-10, and it remains to be seen whether further studies of this drug will indeed show efficacy, or if a different target within the IL-10 activation pathway may prove to be more appropriate.

While there are no studies, to our knowledge, supporting direct effects of androgens on the development of Bregs, testosterone is known to suppress B cell expansion in general (37, 38), and may hence suppress Bregs as well. Alternatively, testosterone may drive Breg differentiation indirectly via effects of other cells involved in the differentiation and/or maintenance of Bregs. For example, a study by Olsen found that bone marrow stromal cells were required, and mediated the B cell suppressive effects of androgens through TGF-β secretion (39). Thus, in this case androgens exerted their effect primarily on pro-B cell populations centrally, with little effect on peripheral cells, potentially redirecting differentiation of B cells toward a more suppressive phenotype as well. Testosterone have also been found to directly regulate BAFF levels, a key mediator of B cell development and maintenance (40). Using a scleroderma model, Matsushita et al. found that BAFF suppressed Bregs, while a BAFF antagonist reduced B effector cells but did not significantly alter Bregs (41), thus providing a molecular mechanism for a skewing toward Bregs in males. Further studies are needed to elucidate if other mechanisms also facilitate a role for testosterone in B cell maturation. Belimumab, an antibody against BAFF, is currently FDA approved for lupus and has been shown to have some effect in SLE patients, altering multiple B cell subsets (42). While data specifically on Breg levels before/after treatment have not been reported, there appears to be alterations in expression of some of the markers for Bregs (42). A number of other anti-BLys and anti-APRIL drugs have been studied (Atacicept, Blisibimod, Tabalumab), but have not seen the same level of success (43, 44). There is an ongoing study to investigate the pathways in which BAFF and APRIL act in order to identify specific patient populations that would be ideal candidates for belimumab among lupus patients (NCT03919643). It would be interesting to see if this trial finds any differences in Breg levels and if gender or hormone levels associate with response to therapy.

### M2 Macrophages

Macrophages have diverse phenotypes and can be distinguished as inflammatory M1 macrophages and anti-inflammatory/repair M2 macrophages (45). While M1 macrophages are known to be pathogenic in lupus, recent studies have suggested a role for M2 macrophages in damping the immune response (46, 47). As such, adoptive transfer of M2 resulted in a reduction in SLE severity in a mouse model (48). Therapeutically, PAM3, a TLR2/1 agonist, has been shown to skew macrophages toward a M2 phenotype in lupus mouse models (49), however the use of PAM3 as a therapeutic agent is controversial and may likely spike unwanted immune activation.

Testosterone acts on macrophages via androgen receptors (50, 51), however few studies have evaluated testosterones effect specifically on M2 macrophages. In one example, Ma et al. found that blocking the androgen receptor alleviated inflammation and promoted M2 polarization in a myocarditis *in-vitro* model (52). Similarly, a recent study by Zhu found that blocking the AR in renal cells led to decreased kidney stones and increased M2 levels (53), suggesting that androgen receptors exert proinflammatory functions in some disease settings.

Oppositely, among alveolar macrophages (AM) from asthma patients, dihydrotestosterone reduced lung inflammation, and enhanced M2 polarization of AM, despite the finding that M2 macrophages were previously found to correlate with asthma severity (54). While the phenotype of M2s within the alveolar space may not be representative to M2s involved in autoimmune pathologies, the finding suggests that among a more heterogeneous population of M2s, androgens may play a specific immunosuppressive stimulatory role. Finally, as mentioned above, testosterone reduces BAFF secretion (40) and when the BAFF antagonist BAFF-Trap was used in a rheumatoid arthritis model, animals exhibited reduced numbers of DCs but increased levels of not only Tregs and Bregs, but also M2 macrophages (55). Thus, BAFF, as a key regulator of lupus pathogenesis may affect multiple regulatory pathways, and be regulated in part by testosterone. In summary, the role of androgens in M2 activation/suppression remains unclear and more research is needed to unravel both direct and indirect effects.

### Myeloid-Derived Suppressor Cells (MDSCs)

Myeloid derived suppressor cells are a heterogeneous population of immature myeloid cells, which have been shown to suppress immune functions during inflammatory conditions. The cells have been shown to have deleterious effects on cancer [reviewed in (56)], but may hold therapeutic potential in suppressing autoimmune disorders. However, results on their function in autoimmunity have been conflicting, owing likely to the diverse cell subpopulations included within the population of MDSCs (57).

Using the NZB/NZW F1 mouse model of lupus, we found previously that MDSCs are significantly increased in male mice in a testosterone dependent fashion (58). Moreover, *in vitro* studies showed that MDSCs were functionally suppressive in post-pubertal male, but not female, mice, and that *in vivo* depletion of these cells resulted in elevated autoantibody production in males, but not females (58). Further analyses showed that Gr1^+^ cell-depleted male NZB/NZW F1 mice displayed expanded populations of splenic germinal center B cells, plasma cells and T follicular helper cells, critical for driving an antibody response, as well as significantly elevated levels of IL-10 (58, 59). Subsequent studies by Bird et al. in female NZB/NZW F1 mice corroborated these studies and showed that early depletion of MDSCs accelerated disease development, while later depletion had no effect (60). Also, a study of the Sanroque mouse model of lupus displayed a similar phenotype with MDSCs inhibiting germinal center B cells, plasma cells and T follicular helper cells, while promoting IL-10-producing B cells (61) (see Figure 1).

MDSCs have been found to be subject to differentiation to pro-inflammatory dendritic cells or macrophages in response to inflammatory cytokines such as TNFα, IL-6, and IFNγ (62, 63). Given the highly inflammatory milieu of female NWB/NZW F1 mice, including elevated levels of serum GM-CSF, IL-6, and IFNα, it is possible that naturally occurring MDSCs in female lupus-prone mice are induced to differentiate into effector cells with an immune-stimulatory phenotype (64, 65). Alternatively, the inflammatory milieu may promote MDSC-dependent extracellular trap formation (and hence MDSC-driven access to nuclear antigens), as suggested by Vlachou et al. (66), although more studies are needed to demonstrate conditions conducive for this change. Interestingly, although studies of MDSCs in SLE patients are few, it has been suggested that MDSCs (as identified by surface markers) from female SLE patients are pathogenic via the production of reactive oxygen species (64, 65). No studies have yet specifically evaluated levels of MDSCs in male SLE patients and matched healthy controls.

## Testosterone and pDC-Derived IFNα as Regulators of Immunosuppressive Cells?

Similarly to a role for IFNα in inhibiting Tregs and Bregs (see above), IFNα-driven IRF7 was shown to negatively control granulocytic MDSC levels (67), suggesting that IFNα blocks MDSC development (see Figure 1). Plasmacytoid dendritic cells (pDCs) are known for their production of IFNα and IL-6 and their role during anti-viral responses. Although not uniform among all SLE patients, elevated levels of type I interferons (IFNα) and the presence of an interferon-stimulated gene signature in PBMCs from SLE patients, further suggest a critical role for pDCs in lupus pathogenesis. Furthermore, IFNα has been implicated a pathogenic role in most animal models of lupus [reviewed in Zhuang et al. (68)] and IL-6 have repeatedly been associated with disease incidence and activity in NZB/NZW F1 mice (69, 70). Interestingly, production of IFNα and IL-6 can be induced by the activation of toll like receptors 7 and 9 on pDCs in response to RNA/DNA containing immune complexes (71), commonly found in SLE serum. IL-6 can also be produced by monocytes, and an *in vitro* study of SLE-derived PBMCs, showed a down-regulation of IL-6 production in response to testosterone treatment (72).

As a product of hematopoiesis, pDCs are likely subject to regulation by sex hormones, although to our knowledge, this has not been specifically evaluated in SLE patients. In healthy adults, however, female-derived pDCs have been shown to produce significantly more IFNα after *in vitro* TLR7 stimulation than male-derived pDCs, although no difference in the number of these cells was found between the sexes (71). Similarly, *in vitro* studies of pDCs isolated from human infants showed significantly lower IFNα production by male cells than by female cells, a pattern that was even more pronounced if the cells were pretreated with dihydrotestosterone prior to simulation (73).

## Discussion

Overall, our understanding of lupus development has improved over the years, but work remains so that better targeted therapies can be developed. The studies discussed in this review highlight that while testosterone itself is unsuitable as a therapeutic agent, downstream targets of androgens represent *de novo* areas for research. Given the clear suppressive effects of androgens on the immune system, of particular interest is pinpointing direct steps in lupus pathogenesis at which testosterone acts, as these may ultimately become areas at which to target new therapies that would maximize disease control while minimizing unwanted side effects. Of particular interest is understanding the mechanism(s) through which testosterone affects numbers and functionality of immunoregulatory cells such as MDSCs, Tregs, Bregs, and M2 macrophages to maintain immunosuppression in genetically predisposed male mice. For example, the direct effect of testosterone on pDCs and IFNα production in combination with testosterone-driven myelopoiesis may directly affect MDSC and M2 skewing, while direct binding to the Foxp3 promoter may regulate Tregs and downregulation of BAFF may affect Breg levels and functions in SLE patients and lupus models exhibiting elevated IFNα levels (see Figure 1). While therapies targeting pDCs and IFNα, along with autologous Treg treatment, are currently being investigated in SLE patients, MDSCs and Breg are being actively studied as a therapeutic target in a number of cancer treatments but not in SLE. We propose that these cells, in their native immunosuppressive state, should also be evaluated for their therapeutic potential in lupus and other autoimmune disorders.

## Author Contributions

All authors listed have made a substantial, direct and intellectual contribution to the work, and approved it for publication.

## Conflict of Interest

The authors declare that the research was conducted in the absence of any commercial or financial relationships that could be construed as a potential conflict of interest.
